# Multimodality Imaging in an Adult Patient with Scimitar Syndrome

**DOI:** 10.4274/mirt.43531

**Published:** 2014-06-05

**Authors:** Alper Özgür Karaçalıoğlu, Seyfettin Gümüş, Semra İnce, Sait Demirkol

**Affiliations:** 1 Gülhane Military Medical Academy, Department of Nuclear Medicine, Ankara, Turkey; 2 Gülhane Military Medical Academy, Department of Pulmonary Medicine, Ankara, Turkey; 3 Gülhane Military Medical Academy, Department of Cardiology, Ankara, Turkey

**Keywords:** Scimitar syndrome, Tc-99m MIBI, scintigraphy, imaging

## Abstract

The “Scimitar syndrome” is a rare congenital anomaly characterized by combination of partial or complete pulmonary venous return from the right lung to the inferior vena cava either above or below the diaphragm together with hypoplasia of the right lung and sometimes systemic arterial supply to the right lung. In this case, multimodality imaging findings such as the vein draining into the inferior vena cava, the presence of hypertrophied and dilated right ventricle, the absence of other cardiac abnormalities, displacement of the heart without malrotation and the mediastinum to the right, normal bronchial and vascular continuity in the whole lung, absence of pulmonary sequestration and systemic collaterals, normal perfusion and systolic functions of the left ventricle were reported.

## Abstract (tr)

Scimitar sendromu, sağ akciğer pulmoner venöz dönüşün bir kısmının veya tamamının diyafragmanın altından ya da üstünden vena kava inferiora drenajı ile birlikte sağ akciğer hipoplazisi ve sağ akciğere sistemik kollateral varlığı ile karakterize nadir konjenital bir anomalidir. Bu vakada, inferior vena kavaya drene olan sağ pulmoner veni, mediasten ve kalbin sağa deviyasyonunu, tüm akciğerin korunmuş vasküler ve bronşiyal bütünlüğünü, akciğere sistemik kollaterallerin yokluğunu, pulmoner sekestrasyonun ve diğer kardiyak anomalilelerin yokluğunu, hipertrofik ve dilate sağ ventrikül ile normal perfüzyon ve sistolik fonksiyonları olan sol ventrikülü gösteren, farklı görüntüleme yöntemlerinden elde edilen bulgular sunulmuştur.

## INTRODUCTION

The “Scimitar syndrome” is a rare congenital anomaly characterized by combination of partial or complete pulmonary venous return from the right lung to the inferior vena cava either above or below the diaphragm together with hypoplasia of the right lung and sometimes systemic collateral vessels to the right lung (1,2,3). Descending vein toward the diaphragm on the right of the heart produces a curvilinear avascular shadow, resembling a curved Turkish sword named “scimitar” on the chest radiograph, and therefore the syndrome derives its name from this appearance (2). The insults causing this malformation in early embryogenesis are unknown. According to the starting of symptoms during infancy or childhood-adulthood, two forms of Scimitar syndrome have been reported (4,5). The infantile form is a severe form of the disease which has a worse prognosis, and has a presentation with major associated cardiac lesions, varying degrees of hypoplasia of the right pulmonary artery and right lung bronchial anomalies, dextroposition of the heart, pulmonary hypertension, and anomalous systemic supply to the right lung. The diagnosis of adult form is made incidentally, and indeed, some patients may be completely asymptomatic. 

Although, the surgical management of Scimitar syndrome is controversial, partial or total resection of the right lung, division of the abnormal arterial supply to the right lung, or redirecting the scimitar vein into the left atrium may be effective treatment options (6). The age at the time of detection of Scimitar syndrome and the presence of associated anomalies is important in predicting the outcome. In contrast to better prognosis of those with the adult form with or without surgery, patients with infant scimitar generally have a poor prognosis, even with aggressive surgical management. 

## CASE REPORT

**Figure 1:**
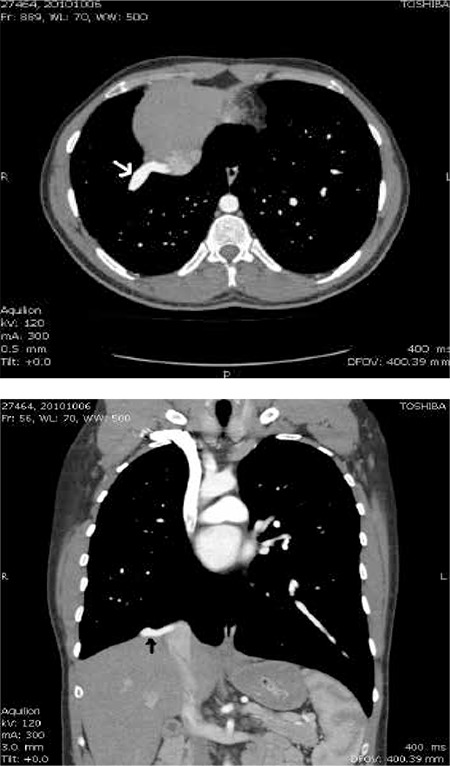
Axial (A) and reconstructed coronal (B) contrast-enhanced CT scans illustrate the anomalous vein draining into the inferior vena cava (arrows). Associated findings are decreased aeration of the right lung and mediastinal shift to the right.

**Figure 2:**
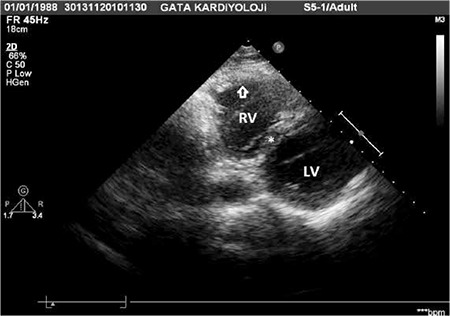
Transthoracic echocardiogram from left parasternal, basal, short axis projection also demonstrates hypertrophied [wall thickness (arrow): 11.4 mm] and dilated right ventricle (18.1 cm2). LV: Left ventricle, RV: Right ventricle, Asterisk: Interventricular septum.

**Figure 3:**
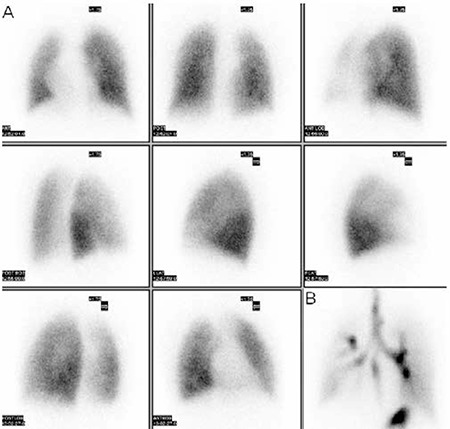
(A) Planar perfusion images of the lung in different projections demonstrate decreased perfusion of the right lung and displacement of the heart and mediastinum to the right. Any perfusion defect to be a sign of systemic arterial perfusion is not detected. (B) Ventilation scan in anterior projection demonstrates reduced volume of the right lung with mediastinal shift to the right side, but it also reveals the bronchial continuity in the lung.

**Figure 4:**
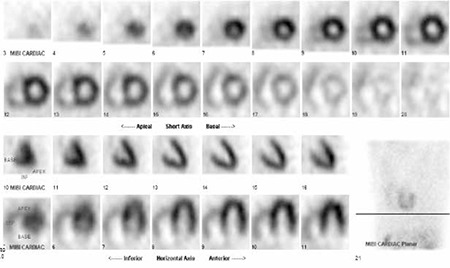
Stress gated SPECT images show the right ventricular dilatation and prominent radiopharmaceutical uptake in the wall of the right ventricle indicating wall hypertrophy together with normal left ventricular myocardial perfusion.

Twenty-year-old man with Scimitar syndrome was referred to our department for myocardial perfusion imaging due to complaints of chest pain. The diagnosis of the syndrome was made by contrast-enhanced computed tomography (CT) in this patient. Transaxial (Figure 1a) and reconstructed coronal (Figure 1b) CT scans of the patient illustrated the vein draining into the inferior vena cava. Transthoracic echocardiogram was performed to evaluate the volume overload of right ventricle in the patient, and it demonstrated the presence of hypertrophied (wall thickness: 11.4 mm) and dilated right ventricle (18.1 cm2) (Figure 2). Besides, the absence of other cardiac abnormalities that may be associated with the syndrome was also confirmed by this modality. Lung perfusion and ventilation scans were acquired to evaluate presence of sequestration in the right lung. After the intravenous injection of macroaggregated albumin (~400.000 particles) labeled with 5 mCi (185 MBq) Tecnetium-99m, decreased perfusion of the right lung indicating hypoplasia and displacement of the heart without rotation of the apex and the mediastinum to the right (Figure 3a) were noticed on planar images recorded in standard eight different projections. Ventilation scan with Tc-99m DTPA aerosols in anterior projection showed normal bronchial communication in the whole lung (Figure 3b) and probability of sequestration was eliminated. Thirty minutes after the injection of Tc-99m MIBI at a dose of 20 mCi (740 MBq) during the peak exercise, stress gated SPECT imaging of the heart was performed by using standard acquisition protocol. Although perfusion and systolic functions of the left ventricle (End-diastolic left chamber volume was 125 ml, end-systolic left chamber volume was 49 ml, ejection fraction was 61%) were normal, right ventricular cavity dilatation and right ventricle wall hypertrophy resulting from chronic right heart overload (Figure 4) were noticed on SPECT scans. 

## LITERATURE REVIEW AND DISCUSSION

The name of the syndrome was first mentioned by Halasz, et al. in 1956 and by Neills et al. in 1960 due to appearance of the anomalous vein on chest X-ray, but the anatomic abnormalities of Scimitar syndrome were described for the first time by Drs. Chassinat and Cooper in 1836 (1,7). In most cases of Scimitar syndrome, partial or complete anomalous drainage of the right lung is provided by abnormal vein that courses inferiorly and medially before passing through the diaphragm and draining into the upper inferior vena cava. In addition to classic findings of Scimitar syndrome, this condition is often associated with other anomalies including dextroposition of the heart, bronchopulmonary sequestration, right lung hypoplasia, hypoplastic or absent pulmonary artery, and anomalous systemic arterial supply to the right lung and other cardiovascular abnormalities. Although the incidence is reported to be approximately 1 to 3 per 100.000 live births (8), the true incidence may be higher due to asymptomatic patients with adult form of this syndrome. Different forms of Scimitar syndrome have been described in the medical literature and it has a variable presentation based on the age at which the diagnosis is made (4,5). The most severe form is in the infantile form, because usually developing severe pulmonary hypertension with respiratory insufficiency and cardiac failure cause patients to become symptomatic within the first year of life. In the other infantile form of Scimitar syndrome, the pulmonary venous return anomaly is usually associated with other cardiothoracic abnormalities including other vascular malformations, hypoplastic left ventricle, atrial or ventricular septal defects, tetralogy of Fallot’s, abnormalities of the aortic arch and abnormal relationship of the pulmonary arteries and bronchi. In the adult form of Scimitar syndrome, patients are usually asymptomatic and the diagnosis is often made incidentally as in our case. The most frequent symptoms of adult-form Scimitar include repeated episodes of pneumonia, minor dyspnea on exertion and occasionally hemoptysis. Pulmonary hypertension is extremely rare in these patients, and if present, is mild and not associated with right heart failure. Although the pulmonary hypertension was not detected in our case, right ventricular dilatation and wall hypertrophy were noticed on SPECT scans due to increased workload caused by a shortcut between the right side of the heart and the right pulmonary. Besides, the problems related to position or rotation of the heart such as mesocardia or dextrocardia may accompany to the syndrome due to the hypoplasia of the right lobe. Hence, the start angle of the SPECT should be adjusted before the acquisition in order to decrease the attenuation artifacts. Since it is usually discovered as an incidental finding in the adult form (9), the syndrome should be considered when there is a prominent activity in the walls of dilated right ventricle on myocardial perfusion imaging especially in young adults. 

The diagnosis is usually established on chest radiograph that shows dextroposition of the heart and hypoplasia of the right lung. Definitive diagnosis is based on the demonstration of the scimitar vein and is usually made by computerized tomography, magnetic resonance imaging and/or angiography in these patients (10,11,12). These modalities allow for direct visualization of the anomalous vein, identification of associated malformations and measurement of shunt. The physiological determinants of the severity of syndrome include the extent of the right-sided volume overload, amount of systemic to venous shunting, and complex interaction between the deviated airway and great vessels. If surgery is considered, ventilation/perfusion scans are recommended as a routine by some investigators both for initial evaluation and follow-up, because ventilation perfusion scans can help a preoperative diagnosis of systemic arterial supply to the lung with or without sequestration (13). Although pulmonary sequestration causes matched perfusion and ventilation defects, systemic arterial supply to the lung without sequestration causes only perfusion defects (14), and therefore, if any perfusion defect is detected in a case of Scimitar syndrome, the probability of systemic arterial supply to the lung should be taken into account. Besides, detection of perfusion on the lung perfusion scan of the patient with this syndrome can eliminate suspicion of right pulmonary artery agenesis. Since there was not any perfusion defect on the lung perfusion and ventilation scan of our patient, the patency of the right pulmonary artery and absence of systemic arterial supply to the right lung were demonstrated in a single test. Swyer-James syndrome and lung hypoplasia should be considered in the differential diagnosis of this syndrome, and therefore, ventilation/perfusion scans of the lung can be helpful by demonstrating the bronchovascular continuity. 

Since patients with the adult form of the syndrome are asymptomatic and diagnosis is made incidentally, the multimodality imaging seems to be helpful to discover new asymptomatic cases and management of patients with this syndrome.
